# TGFB-induced factor homeobox 1 (TGIF) expression in breast cancer

**DOI:** 10.1186/s12885-021-08656-0

**Published:** 2021-08-14

**Authors:** Christine Stürken, Volker Möbus, Karin Milde-Langosch, Sabine Schmatloch, Peter A. Fasching, Josef Rüschoff, Elmar Stickeler, Rolf-Peter Henke, Carsten Denkert, Lars Hanker, Christian Schem, Valentina Vladimirova, Thomas Karn, Valentina Nekljudova, Claus-Henning Köhne, Frederik Marmé, Udo Schumacher, Sibylle Loibl, Volkmar Müller

**Affiliations:** 1 Department of Gynecology, University Medical Center Hamburg-Eppendorf Martinistrasse 52, 20246 Hamburg, Germany; 2 Klinik für Gynäkologie und Geburtshilfe, Klinikum Frankfurt Höchst GmbH, Frankfurt am Main, Germany; 3 Elisabeth Krankenhaus Kassel, Kassel, Germany; 4 Universitätsklinikum Erlangen, Erlangen, Germany; 5 Uniklinik RWTH Aachen, Aachen, Germany; 6 Klinikum Oldenburg, Oldenburg, Germany; 7 Institute of Pathology, Philipps-University Marburg and University Hospital Marburg (UKGM), Marburg, Germany; 8 Department of Gynecology and Obstetrics, University Hospital of Schleswig-Holstein, Campus Lübeck, Kiel, Germany; 9 Mammazentrum Hamburg, Hamburg, Germany; 10 German Breast Group, Neu-Isenburg, Germany; 11 Goethe University, Frankfurt, Germany; 12 Medizinische Fakultät Mannheim, Universität Heidelberg, Universitätsfrauenklinik Mannheim, Mannheim, Germany

**Keywords:** Breast cancer, TGFB-induced factor homeobox 1, TGIF, Bone metastases

## Abstract

**Background:**

Breast cancer (BC) is the most frequent female cancer and preferentially metastasizes to bone. The transcription factor TGFB-induced factor homeobox 1 (TGIF) is involved in bone metabolism. However, it is not yet known whether TGIF is associated with BC bone metastasis or patient outcome and thus of potential interest.

**Methods:**

TGIF expression was analyzed by immunohistochemistry in 1197 formalin-fixed, paraffin-embedded tissue samples from BC patients treated in the GAIN (German Adjuvant Intergroup Node-Positive) study with two adjuvant dose-dense schedules of chemotherapy with or without bisphosphonate ibandronate. TGIF expression was categorized into negative/low and moderate/strong staining. Endpoints were disease-free survival (DFS), overall survival (OS) and time to primary bone metastasis as first site of relapse (TTPBM).

**Results:**

We found associations of higher TGIF protein expression with smaller tumor size (*p* = 0.015), well differentiated phenotype (*p* < 0.001) and estrogen receptor (ER)-positive BC (*p* < 0.001). Patients with higher TGIF expression levels showed a significantly longer disease-free (DFS: HR 0.75 [95%CI 0.59–0.95], log-rank *p =* 0.019) and overall survival (OS: HR 0.69 [95%CI 0.50–0.94], log-rank *p* = 0.019), but no association with TTPBM (HR 0.77 [95%CI 0.51–1.16]; *p* = 0.213). Univariate analysis in molecular subgroups emphasized that elevated TGIF expression was prognostic for both DFS and OS in ER-positive BC patients (DFS: HR 0.68 [95%CI 0.51–0.91]; log-rank *p* = 0.009, interaction *p* = 0.130; OS: HR 0.60 [95%CI 0.41–0.88], log-rank *p* = 0.008, interaction *p* = 0.107) and in the HER2-negative subgroup (DFS:HR 0.67 [95%CI 0.50–0.88], log-rank *p* = 0.004, interaction *p* = 0.034; OS: HR 0.57 [95%CI 0.40–0.81], log-rank *p* = 0.002, interaction *p* = 0.015).

**Conclusions:**

Our results suggest that moderate to high TGIF expression is a common feature of breast cancer cells and that this is not associated with bone metastases as first site of relapse. However, a reduced expression is linked to tumor progression, especially in HER2-negative breast cancer.

**Trial registration:**

This clinical trial has been registered with ClinicalTrials.gov; registration number: NCT00196872.

**Supplementary Information:**

The online version contains supplementary material available at 10.1186/s12885-021-08656-0.

## Background

Breast cancer (BC) is not only the leading cancer among women in every European country but is also the leading cause of death from cancer in women in Europe. Declines in BC mortality rates in most European countries have been reported, the favorable trends result from the combined effects of earlier detection (partly due to screening, partly due to increasing BC awareness), and a range of improvements in treatment [1]. However, the majority of deaths are not due to the primary tumor itself, but are the result of distant metastases to other organs in the body [2]. Metastatic BC remains an incurable disease with a median survival of approximately 20 months. Long-term survivors do exist but are very rare [3]. The most common metastatic site is bone with 60–70% of all metastatic BC patients [4]. Bone metastases are largely incurable and associated with significant morbidity that negatively impacts the quality of life in metastatic BC patients. The development of BC bone metastases is a complex process involving crosstalk between disseminated BC cells and bone-derived molecules, leading to deregulation of signaling pathways critical for normal bone remodeling processes.

The GAIN study is to our knowledge the first randomized clinical trial examining oral ibandronate as adjuvant treatment for patients with early-stage breast cancer (von Minckwitz et al. JCO 2013). Bisphosphonates are pyrophosphate analogs which bind hydroxyl apatite in bone thus inhibiting osteoclast activity to restrict the progression of bone destruction and increase survival [5]. Currently, intravenous bisphosphonates have been the mainstay of the prevention of local irreversible skeletal-related events (SRE) in patients with metastatic solid tumors. From the clinical perspective, there is a high need for a better understanding of the effects from adjuvant bisphosphonate treatment. Studies evaluating the benefit of adjuvant bisphosphonate use revealed conflicting results [6] and therefore predictive/prognostic factors for the benefit of adjuvant bisphosphonate use are of high clinical relevance [7].

Bone formation is strongly activated by the canonical Wnt signaling pathway [8]. A TGFB-induced factor homeobox 1 (TGIF) was identified as a novel Wnt target gene and a crucial regulator of osteoblast function [9]. TGIF is a member of the three-amino acid loop extension (TALE) superclass of atypical homeodomain proteins, a family of highly conserved transcription regulators. The first two helices of the TALE superfamily are separated by a loop, which is likely to affect interactions with other proteins but not alter DNA binding properties [10, 11].

Increasing evidence suggests that TGIF is associated with the initiation, development and progression of several human tumor entities like gastric [12], colorectal [13] and lung cancer [14, 15]. Preliminary results in a small well-characterized primary breast cancer cohort (*n* = 198) showed that a high TGIF mRNA expression detected by microarray analysis was an independent predictor of longer DFS and OS, and associations of low TGIF mRNA levels with bone and brain metastases were found (unpublished data; supplementary Figure 1). Therefore, we aimed to investigate the TGIF protein expression and its association with clinical outcomes in a large cohort of patients with early breast cancer who received adjuvant chemotherapy.

To address the potential role of TGIF in human BC, also in the context of bone metastases, we analyzed TGIF protein expression by immunohistochemistry in 1197 human BC samples using a tissue microarray prepared from formalin-fixed paraffin-embedded (FFPE) tissue samples of BC patients treated with two different dose-dense schedules of adjuvant chemotherapy with or without the bisphosphonate ibandronate.

## Materials and methods

### Patients

The GAIN (German Adjuvant Intergroup Node-Positive) study was a multicenter, prospective, randomized, open-label phase III trial with a 2 × 2 factorial design. Women (aged ≥18 and < 65 years) with involved axillary lymph nodes were randomly assigned to receive three courses each of epirubicin (E) 150 mg/m^2^, paclitaxel (P) 225 mg/m^2^ and cyclophosphamide (C) 2500 mg/m^2^ (reduced to 2000 mg/m^2^ after recruitment of 1200 patients) q2w intravenously (i.v.) (iddEPC-regimen) or ddEC (E 112.5 mg/m^2^ + C 600 mg/m^2^, i.v. q2w for 4 cycles) followed by paclitaxel weekly (Pw 67.5 mg/m^2^ i.v. q8d for 10 weeks) plus capecitabine (X 2000 mg/m^2^ p.o. day 1–14, q22 for 4 cycles) (ddEC-PwX-regimen). Further randomization assigned patients to ibandronate for 2 years vs observation and to pegfilgrastim day 2 vs 4.

Ethical committee approval from all centers participating in the clinical study and from the Institutional Review Board of Charité University Hospital Berlin (Germany; Ethikvotum EA1/139/05) was obtained. All participants had to sign a consent form. This study was conducted adhering to the REMARK (Reporting Recommendations for Tumor Marker Prognostic Studies) criteria [16]. From June 2004 to August 2008, 2994 patients were randomized to either iddEPC (*n* = 1498) or ddEC-PwX (*n* = 1496) and started treatment.

### Immunohistochemistry

The GAIN tissue microarray consists of formalin-fixed paraffin-embedded tissue samples including about 1380 BC tumor samples in 14 paraffin blocks. Freshly cut 4 μm tissue slides were used for immunohistochemistry (IHC). For the detection of TGIF, tissue slides were deparaffinized, and antigen retrieval was performed in citrate buffer solution (pH 6.1) in a steamer for 30 min. Tissue samples were then incubated overnight at 4 C with a rabbit TGIF antibody (Abcam plc, Cambridge, UK; dilution: 1:150) For detection, slides were incubated with biotinylated anti-rabbit secondary antibody and normal goat serum, then ABC Complex (Vectastain, Vector Laboratories) and DAB substrate kit (Vectastain, Vector Laboratories). All slides were counterstained with eosin/hematoxylin. As positive controls, paraffin sections of a BC sample which had been previously shown to stain positive for TGIF were treated in the same way. Omission of the primary antibody served as a negative control.

**Fig. 1:**
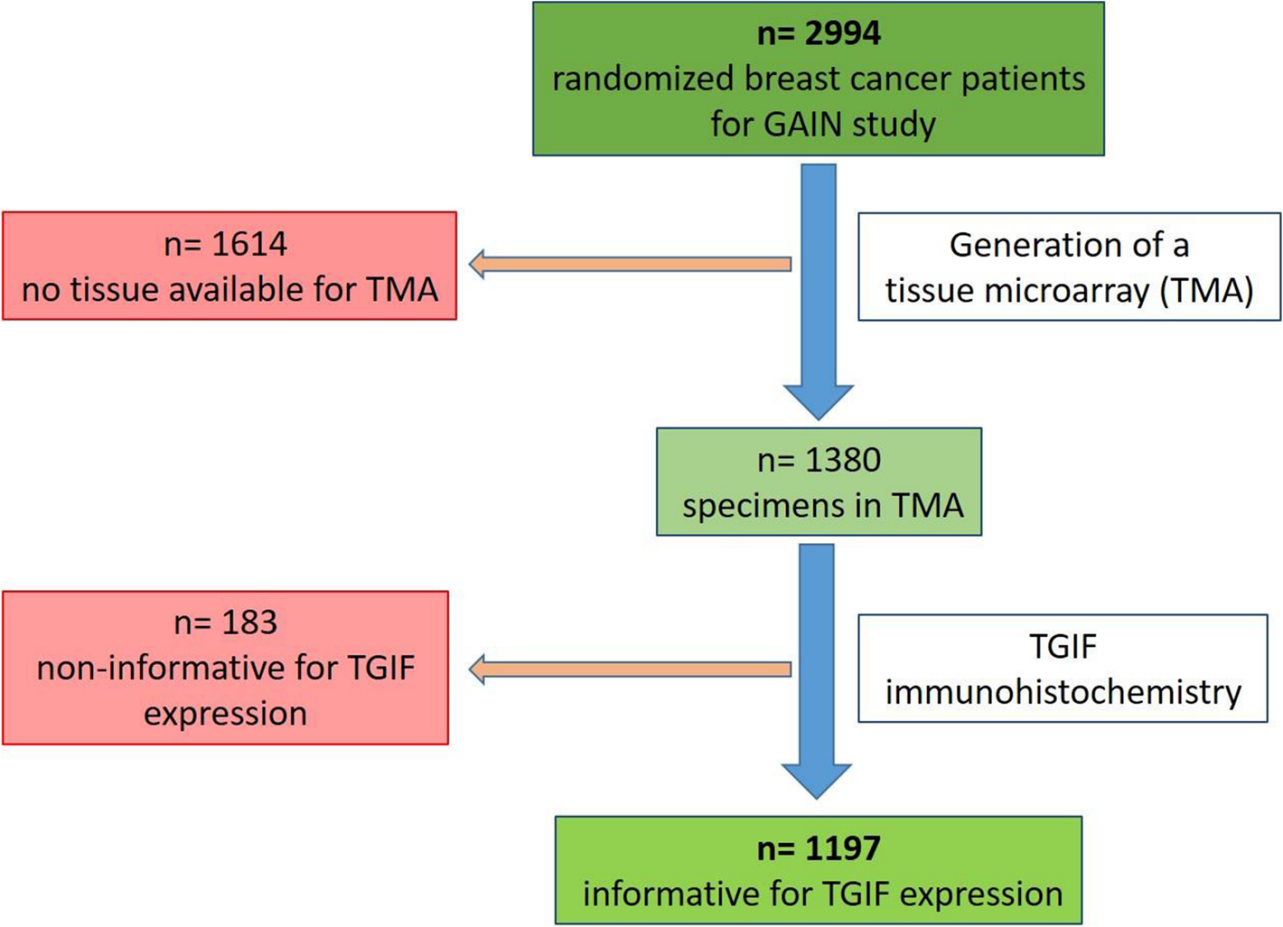
Flow diagram of the technical proceeding

After TGIF immunohistochemistry, 183 (13.3%) tissue specimens were non-informative, due to the lack of tissue or absence of unequivocal cancer cells in the TMA spot. Thus, 1197 cases with evaluable TGIF expression were further analyzed (flow diagram Fig. 1). The staining results were evaluated independently in a blinded fashion by two individuals using the immunoreactive score (IRS) [17] which combines staining intensity and percentage of positive tumor cells resulting in a score of 0–12. For statistical analysis, the expression of TGIF was categorized into negative/low (IRS 0–2) and moderate/strong (IRS 3–12).

### Statistical methods and analysis

Associations between TGIF expression as dichotomized variable with categorical clinical and histological parameters were assessed by Fisher’s exact (two classes) and Pearson Chi-square (three or more classes) test. Survival was analyzed by Kaplan-Meier product-limit method and compared between groups using the log-rank test. Median-follow-up time was estimated with the inverse Kaplan-Meier method. Cox proportional hazard model was performed to evaluate the potential prognostic value of TGIF for disease-free survival (DFS) and overall survival (OS). Hazard ratios (HR) with 95% confidence interval (CI) were presented. The impact of TGIF on DFS or OS was assessed by univariate Cox regression in subgroups with regards to treatment arm (iddEPC vs. ddEC-PwX), ibadronate treatment (with vs. without), ER status (ER-pos. vs. ER-neg.), HER2 status (HER2-pos. vs. HER2-neg.) and TNBC vs. non-TNBC. The interaction between subgroups was assessed by bivariate Cox regression model. All reported *p*-values were two-sided, and *p* < 0.05 were considered statistically significant.

All time-to-event end points were defined as the time (in months) from random assignment to the event; patients without event were censored at the time of the last contact. Events for DFS were any loco-regional (ipsilateral breast or local/regional lymph nodes) recurrence of disease, any contralateral breast cancer, any distant recurrence of disease, any secondary malignancy, or death as a result of any cause, whichever occurred first. OS was defined as the time since random assignment until death as a result of any cause. Event for time to primary bone metastasis (TTPBM) was any bone metastasis occurred as a first site of relapse; local recurrence, other distant metastases, contralateral BC, secondary malignancies or death were considered competing risks. TTPBM was analyzed using the Gray’s competing risk model [18] and the hazard ratio of TTPBM was assessed using Fine-Gray’s regression model [19].

All statistical analyses were performed using SPSS 25.0 (IBM SPSS Statistics 22) and SAS (version 9.4).

## Results

### Patient characteristics and clinicopathological data

After TGIF immunohistochemistry, 1197 cases with evaluable TGIF expression could be analyzed. Detailed patient characteristics and corresponding clinicopathological data are listed in Table 1. The median age was 49 years (range 23–71 years), the median follow-up was 74.1 months (range 0.0 to 113.7 months). Two hundred seventy-three patients had a recurrence, and 159 patients died within the observation period. In 90 cases bone metastasis was the first site of relapse. Results of the GAIN study were published previously [7, 20].

**Table 1:** Correlation of clinicopathological data of breast cancer patients with TGIF expression levels

Parameter	Category	Overall		TGIF expression				*p*-value
				0–2		3–12		
		N	%	N	%	N	%	
**All patients**		1197		475	39.7%	722	60.3%	
**Age (years)**	< 40	185	15.5%	73	15.4%	112	15.5%	0.756
	40–50	489	40.9	196	41.3%	293	40.6%	
	51–65	496	41.4%	56	48.7%	440	40.7%	
	> 65	27	2.3%	8	1.7%	19	2.6%	
**Tumor stage (pT)**	pT1	394	33.0%	134	28.4%	260	36.1%	0.015
	pT2	665	55.7%	286	60.6%	379	52.6%	
	pT3/4	134	11.2%	52	11.0%	82	11.4%	
	total	1193		472		721		
	missing	4						
**Lymph node status (pN)**	pN1	493	41.2%	181	38.1%	312	43.2%	0.114
	pN2	398	33.2%	159	33.5%	239	33.1%	
	pN3	306	25.6%	135	28.4%	171	23.7%	
**Tumor grading (G)**	G1	39	3.3%	13	2.7%	26	3.6%	< 0.001
	G2	588	49.2%	196	41.4%	392	54.3%	
	G3	569	47.6%	265	55.9%	304	42.1%	
	missing	1						
**Histological type**	ductal	930	77.7%	389	81.9%	541	74.9%	0.009
	lobular	136	11.4%	39	8.2%	97	13.4%	
	others	131	10.9%	47	9.9%	84	11.6%	
**HER2**	positive	248	22.0%	89	20.4%	159	23.0%	0.303
	negative	880	78.0%	348	79.6%	532	77.0%	
	missing	69						
**ER**	positive	886	74.0%	322	67.8%	564	78.1%	< 0.001
	negative	311	26.0%	153	32.2%	158	21.9%	
**PR**	positive	819	68.4%	285	60.0%	534	74.0%	< 0.001
	negative	378	31.6%	190	40.0%	188	26.0%	
**Treatment in mITTset**	iddEPC	597	49.9%	232	48.8%	365	50.6%	0.595
	ddEC-PwX	600	50.1%	243	51.2%	357	49.4%	
**Ibandronate treatment**	with Ibandronate	781	65.2%	307	64.6%	474	65.7%	0.756
	without Ibandronate	416	34.8%	168	35.4%	248	34.3%	

Data are N (valid %) unless otherwise state

*mITT* modified intention-to treat, *HER2* human epidermal growth factor receptor 2, *ER* estrogen receptor, *PR* progesterone receptor, *iddEPC* intense dose-dense epirubicin plus paclitaxel plus cyclophosphamide, *ddEC-PwX* dose-dense epirubicin plus cyclophosphamide plus paclitaxel weekly and capecitabine

### TGIF expression in tumor tissue

**Fig. 2:**
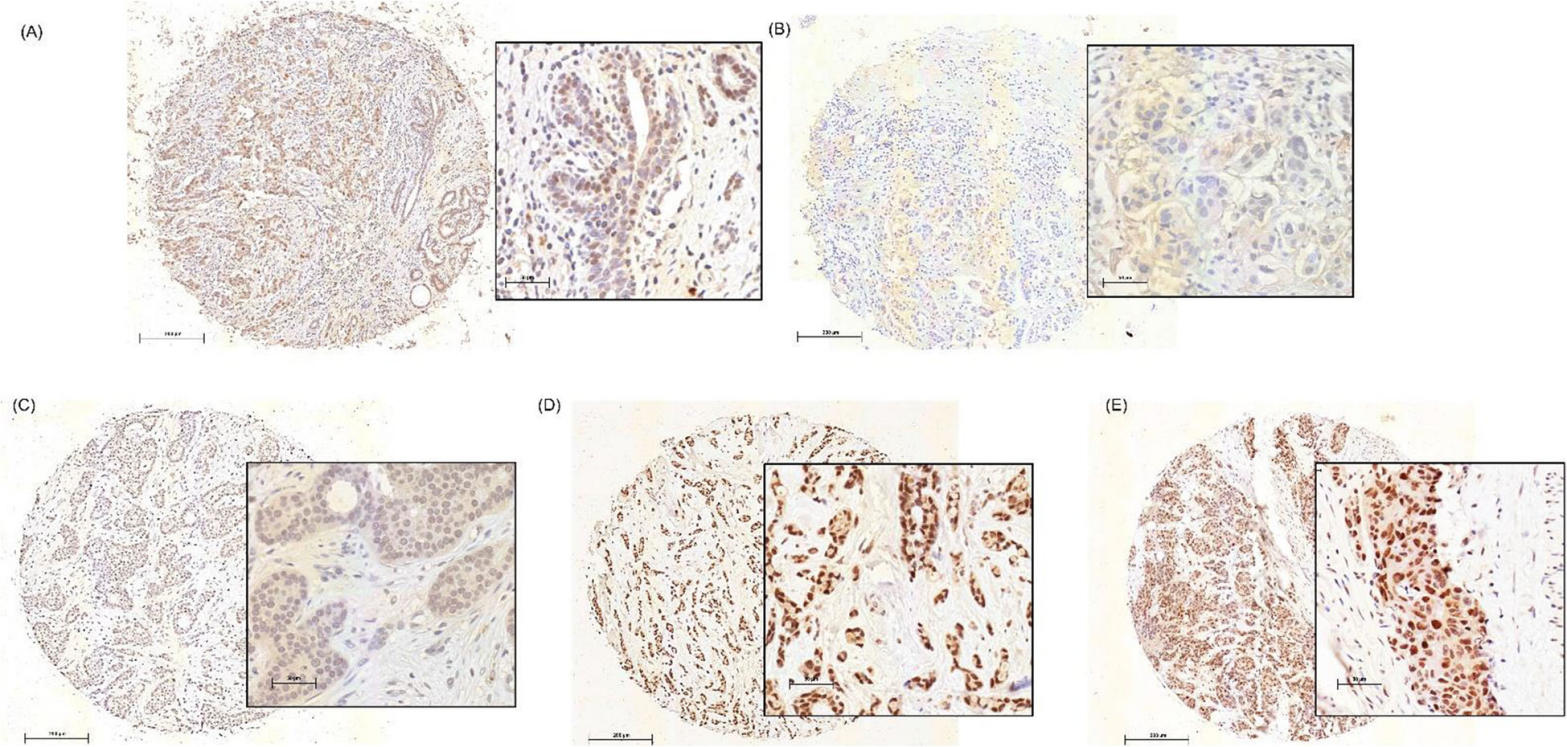
Representative pictures of TGIF immunostaining; **A** Tumor with normal epithelium; **B** negative; **C** low; **D** moderate and **E** strong TGIF expression in breast cancer samples. Scale bar 200 / 50 μm

Weak TGIF immunoreactivity was located in the nucleus and in some cases also in the cytoplasm of normal luminal epithelial cells, if present within the slide (Fig. 2).

In breast carcinoma cells, only nuclear TGIF protein expression was evaluated, although weaker cytoplasmic staining was partly observed (Fig. 2). In 115 cases (9.6%), no TGIF staining was observed in tumor cells. Weak staining (IRS 1–2) was detected in 360 tumor tissues (30.1%), moderate staining (IRS 3–5) in 325 cases (27.2%) and strong staining (IRS 6–12) in 397 cases (33.2%). Representative images of these five groups are presented in Fig. 2. Analyzing TGIF expression as dichotomized variable demonstrated that 475 cases (39.7%) had TGIF-negative/low and 722 cases (60.3%) moderate/strong immunoreactivity.

We found that a moderate/strong TGIF protein expression compared to negative/low TGIF levels was significantly associated with smaller tumor size (36.1% vs 28.4%, respectively; *p* = 0.015) and a well differentiated phenotype (3.6% vs 2.7%; *p* < 0.001; Table 1). Seventy-eight percent (564 cases) of the tumors with higher expression of TGIF were ER-positive, in contrast to 67.8% (322 cases) of the tumors with negative/low TGIF levels (*p* < 0.001). A similar association of TGIF expression and progesterone receptor (PR)-positive tumors was observed (74.0% vs 60.0%, respectively; *p* < 0.001). Furthermore, moderate/strong TGIF expression was less frequently detected in ductal carcinomas than negative/low TGIF expression (74.9% vs 81.9%, respectively; *p* = 0.009). No significant correlations with age, HER2 status or nodal involvement could be detected (Table 1).

### Elevated TGIF expression correlates with longer DFS and OS

**Fig. 3:**
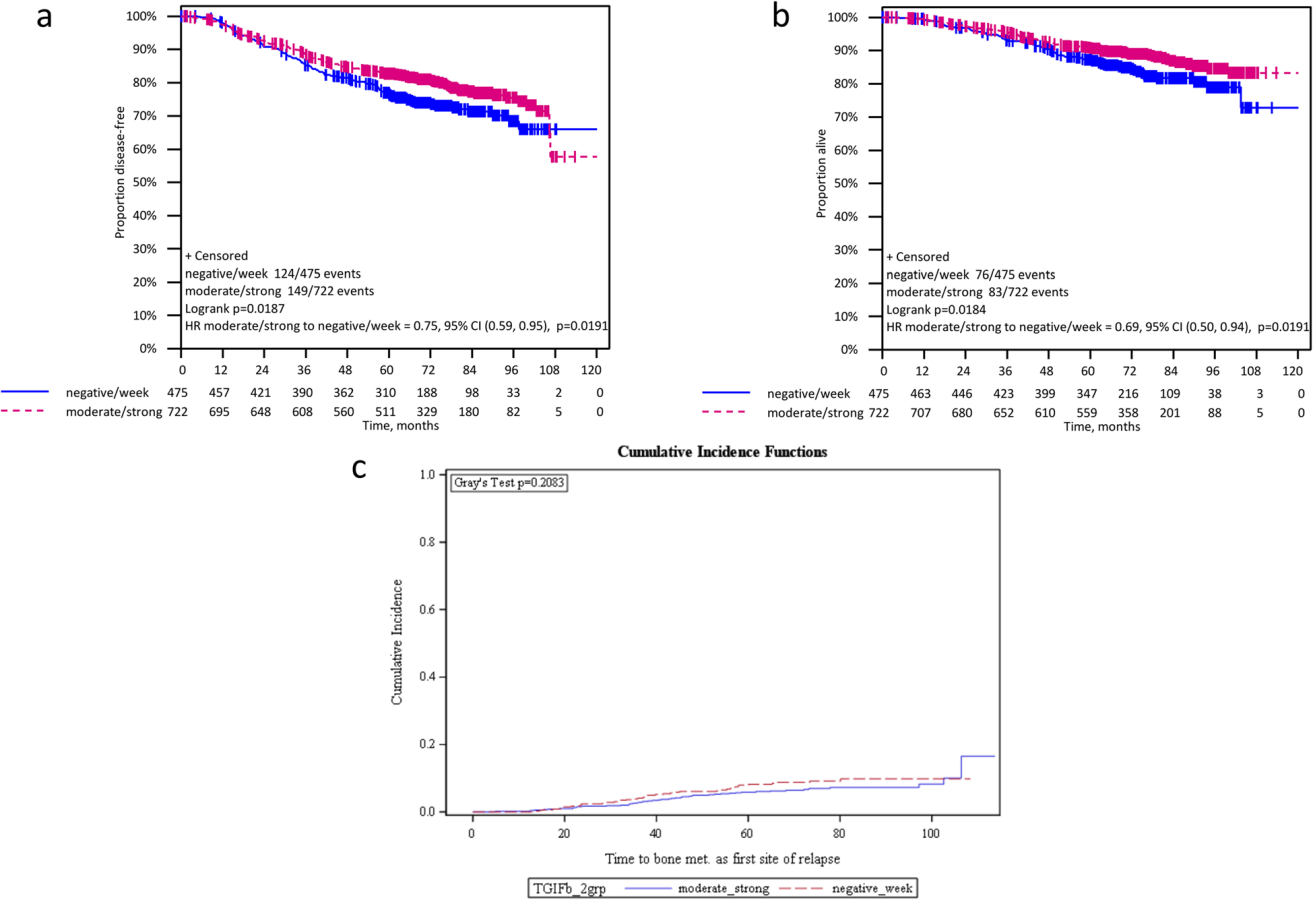
Kaplan-Meier analysis for DFS (**A**) (HR 0.75 [95%CI 0.59–0.95]; log rank *p* = 0.019), OS (**B**) HR 0.69 [95%CI 0.50–0.94]; log rank *p* = 0.019) and TTPBM (**C**) (HR 0.77 [95%CI 0.51–1.16]; *p* = 0.213) according to the overall TGIF immunostaining (moderate/strong vs negative/low). DFS, disease-free survival, OS, overall survival; TTPBM, time to primary bone metastasis

**Fig. 4:**
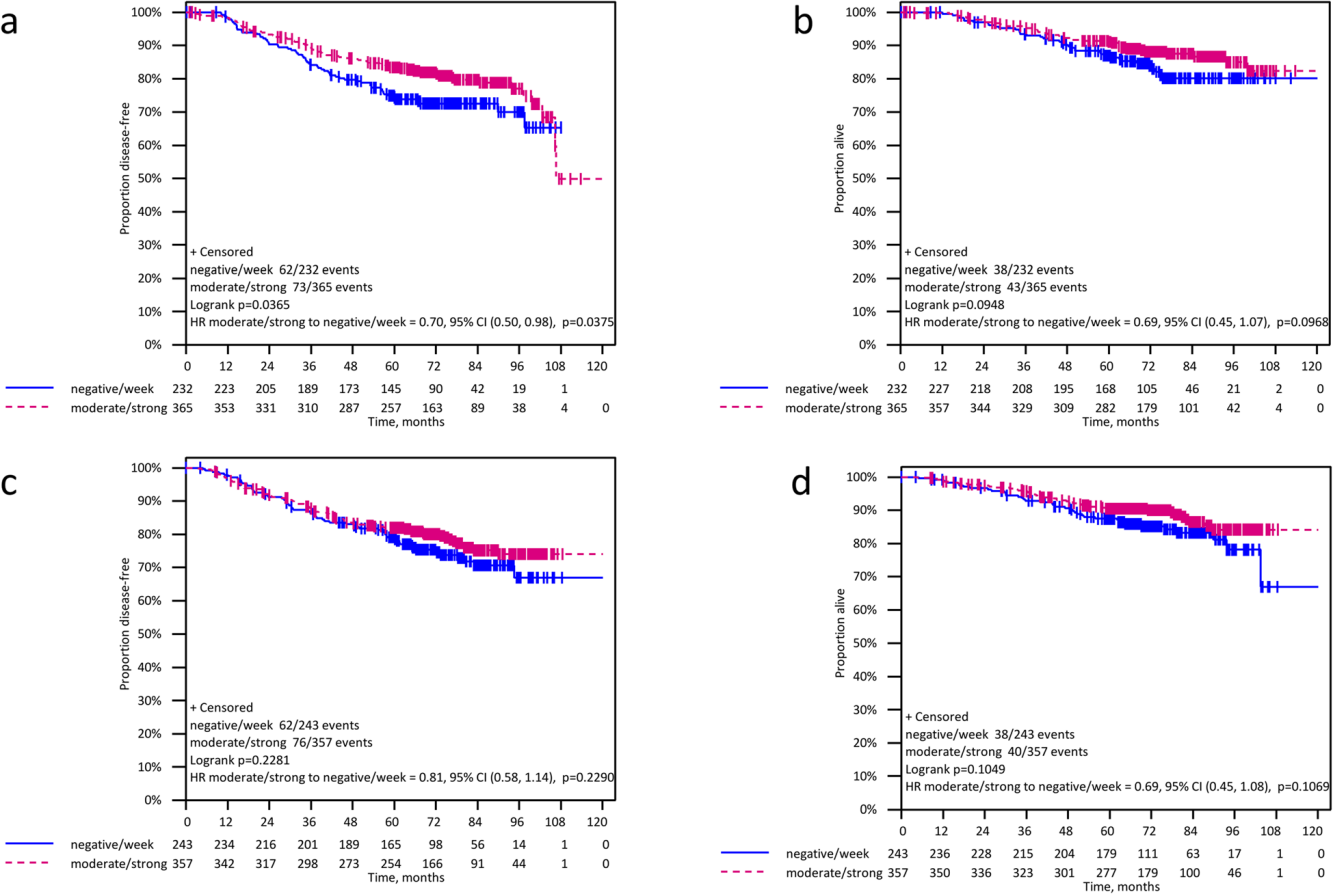
Subgroup analysis for DFS and OS according to the TGIF immunostaining (moderate/strong vs negative/low) with regards to treatment arm (iddEPC and ddEC-PwX): **A** DFS iddEPC treatment (HR 0.70 [95% CI 0.50–0.98]; log-rank *p* = 0.037). **B** OS iddEPC treatment (HR 0.69 [95% CI 0.45–1.07]; log-rank *p* = 0.096). **C** DFS iddEC-PwX treatment (HR 0.81 [95% CI 0.58–1.14]; log-rank *p* = 0.229). **D** OS iddEC-PwX treatment HR 0.69 [95% CI 0.45–1.08]; log-rank *p* = 0.106

After a median follow-up of 74.1 months (range 0.0–113.7) patients with moderate/strong TGIF expression showed a significantly longer DFS (HR 0.75 [95%CI 0.59–0.95]; log rank *p* = 0.019) and OS (HR 0.69 [95% CI 0.50–0.94]; log rank *p* = 0.019) compared to patients with negative/low TGIF expression. For TTPBM as first site of relapse no significant correlation with TGIF expression was found (HR 0.77 [95% CI 0.51–1.16]; *p* = 0.213) (Fig. 3 and Table 2). A subgroup analysis by treatment arm (iddEPC and ddEC-PwX) showed a significant improvement of DFS for patients with moderate/strong TGIF expression compared to those with negative/low TGIF staining in the iddEPC arm (HR 0.70 [95% CI 0.50–0.98]; log-rank *p* = 0.037), but not in the ddEC-PwX arm (HR 0.81 [95% CI 0.58–1.14]; log-rank *p* = 0.229; interaction *p* = 0.575). Regarding OS, the prognostic effect of TGIF expression was not significant in both treatment groups when analyzed separately (iddEPC: HR 0.69 [95% CI 0.45–1.07]; log-rank *p* = 0.096 and ddEC-PwX: HR 0.69 [95% CI 0.45–1.08]; log-rank *p* = 0.106) (Fig. 4, Table 2). Note that the TTPBM analysis in subgroups is not presented due to the small number of bone metastases occurred as first event.

**Table 2:** Analysis and prognostic value of TGIF expression overall and in patient subgroups

Analysis	Parameter	DFS			OS		
		HR^a^ (95%CI)	Log-rank *p*-value	Bivariate interaction *p*-value	HR^a^ (95%CI)	Log-rank *p*-value	Bivariate interaction *p*-value
***Overall***							
	TGIF moderate/strong	0.75 (0.59–0.95)	0.019	–	0.69 (0.51–0.94)	0.018	–
***Subgroups***							
iddEPC	moderate/strong TGIF	0.70 (0.50–0.98)	0.036	0.575	0.69 (0.45–1.07)	0.095	0.978
ddEC-PwX	moderate/strong TGIF	0.81 (0.58–1.14)	0.228		0.69 (0.45–1.08)	0.105	
Ibandronate treatment	moderate/strong TGIF	0.79 (0.59–1.07)	0.131	0.620	0.72 (0.49–1.06)	0.094	0.729
without Ibandronate treatment	moderate/strong TGIF	0.68 (0.46–1.01)	0.057		0.64 (0.38–1.07)	0.087	
ER-negative	moderate/strong TGIF	1.03 (0.67–1.58)	0.893	0.130	1.02 (0.60–1.75)	0.933	0.107
ER-positive	moderate/strong TGIF	0.68 (0.51–0.91)	0.009		0.60 (0.41–0.88)	0.008	
HER2-negative	moderate/strong TGIF	0.67 (0.50–0.88)	0.004	0.034	0.57 (0.40–0.82)	0.002	0.015
HER2-positive	moderate/strong TGIF	1.27 (0.75–2.18)	0.375		1.79 (0.80–4.00)	0.151	
TNBC	moderate/strong TGIF	1.00 (0.57–1.75)	0.995	0.427	1.05 (0.56–1.96)	0.876	0.291
non-TNBC	moderate/strong TGIF	0.76 (0.58–1.00)	0.049		0.70 (0.48–1.01)	0.055	

^a^negative/low TGIF expression is reference

Prognostic value of TGIF for TTPBM, overall: HR = 0.77 (95%CI 0.51–1.16); *p* = 0.213. Of note, the TTPBM analysis in subgroups is not presented due to the small number of bone metastases occurred as first event

*HER2* human epidermal growth factor receptor 2, *ER* estrogen receptor, *PR* progesterone receptor, *iddEPC* intense dose-dense epirubicin plus paclitaxel plus cyclophosphamide, *ddEC-PwX* dose-dense epirubicin plus cyclophosphamide plus paclitaxel weekly and capecitabine, *TNBC* triple-negative breast cancer, *HR* hazard ratio, *DFS* disease-free survival, *OS* overall survival, *CI* confidence interval, *TTPBM* time to primary bone metastasis

### Prognostic role of TGIF is independent of ibandronate treatment

Considering treatment with ibandronate, the prognostic value of moderate/strong TGIF expression did not reach statistical significance for DFS and OS but showed a tendency towards improved survival in the individual groups with or without ibandronate treatment: In patients with ibandronate the HR for DFS was 0.79 (95%CI 0.59–1.07; log-rank *p* = 0.131) and for OS 0.72 (95%CI 0.49–1.06; log-rank *p* = 0.094). Patients without ibandronate treatment showed a HR for DFS of 0.68 (95%CI 0.46 1.01; log-rank *p* = 0.057), as well as a HR for OS of 0.64 (95%CI 0.38–1.07; log rank *p* = 0.087) (Table 2).

### Better prognosis of ER-positive and HER2-negative patients with higher TGIF expression

**Fig. 5:**
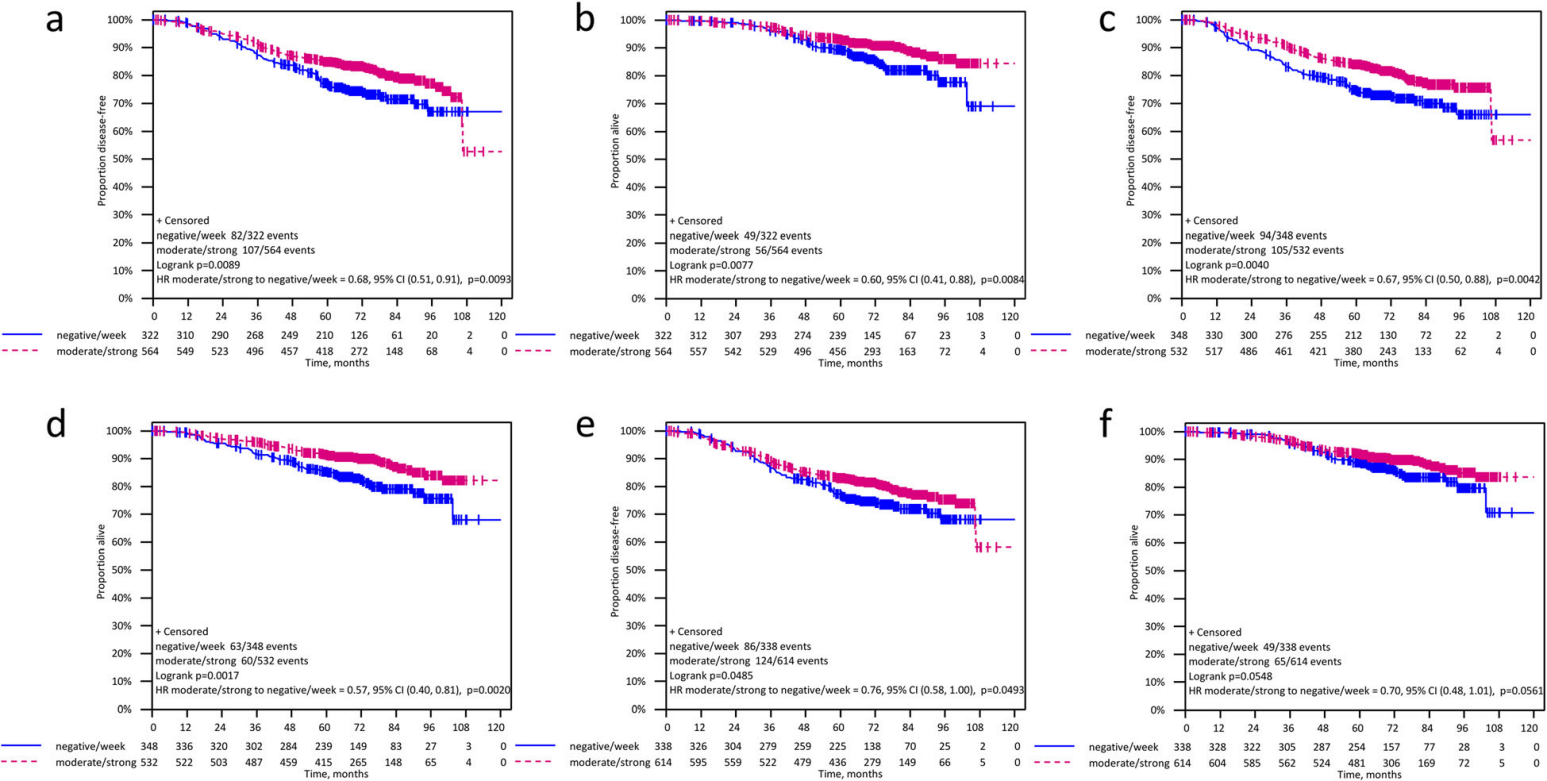
Stratified Kaplan-Meier analysis for DFS and OS by estrogen receptor (ER) status, by HER2 status, and by triple-negative status: **A** DFS in ER-positive BC subgroup (HR 0.68; 95%CI 0.51–0.91; log-rank *p* = 0.009) **B** OS in ER-positive BC subgroup (HR 0.60; 95%CI 0.41–0.88; log-rank *p* = 0.008); **C** DFS in HER2-negative BC subgroup (HR 0.67; 95%CI 0.50–0.88; log-rank *p* = 0.004) **D** OS in HER2-negative BC subgroup (HR 0.57; 95%CI 0.40–0.81; log-rank *p* = 0.002) **E** DFS in non-TNBC subgroup (HR 0.76; 95%CI 0.58–1.00; log-rank *p* = 0.049) **F** OS in non-TNBC subgroup (HR 0.70; 95%CI 0.48–1.01; log-rank *p* = 0.056)

With regards to ER status, the subgroup analysis showed that moderate/strong TGIF expression was significantly prognostic in patients with ER-positive tumors for both DFS (HR 0.68 [95%CI 0.51–0.91]; log-rank *p* = 0.009; interaction *p* = 0.130) and OS (HR 0.60; 95%CI 0.41–0.88; log-rank *p* = 0.008; interaction *p* = 0.107; Fig. 5A-B), in contrast to patients with ER-negative tumors. Yet, the interaction between TGIF expression and ER status was not statistically significant (Table 2). Regarding the HER2 status, in the HER2-negative subgroup, elevated TGIF expression was significantly associated with better DFS (HR 0.67 [95%CI 0.50–0.88]; log-rank *p* = 0.004; interaction *p* = 0.034) and OS (HR 0.57 [95%CI 0.40–0.81]; log-rank *p* = 0.002; interaction *p* = 0.015; Fig. 5C-D) with a significant interaction between TGIF expression and HER2 status (Table 2).

Comparing triple-negative breast cancer (TNBC) patients with non-TNBC patients, we found that only in non-TNBC, moderate/strong TGIF expression was significantly associated with a longer DFS (HR 0.76 [95%CI 0.58–1.00]; log-rank *p* = 0.049; interaction *p* = 0.427) and we could observe a trend towards a better OS (HR 0.70 [95%CI 0.48–1.01]; log-rank *p* = 0.056; interaction *p* = 0.291) (Table 2 and Fig. 5E-F). However, no significant interaction was observed between TGIF expression and triple-negative status.

## Discussion

Cancer related formation of metastasis is a highly complex process and bone is the most frequent site of metastatic relapse in BC. Bisphosphonates are established in the treatment of manifest bone metastases and evidence suggests also the ability of these compounds to prevent bone metastases. However, most studies examining the adjuvant use of bisphosphonates did not reach their endpoint despite a positive trend and a positive meta-analysis [21]. Therefore, our aim was to examine a potential marker for metastases formation that is also potentially prognostic for an adjuvant bisphosphonate effect. Due to its reported role in cancer, TGIF seemed a promising candidate in this respect.

The rationale for our analysis was the observation of TGIF as a novel Wnt target gene and a crucial regulator of osteoblast function: Absence of TGIF impairs osteoblast differentiation in vitro and osteoblast activity and bone formation in vivo [9] and might therefore influence bone metastasis in cancer patients. In our own unpublished study, low TGIF mRNA expression was associated with poor outcome and a higher frequency of bone and brain metastasis (Supplementary Figure 1).

Canonical Wnt signaling is among the most prominent pathways promoting osteoblast differentiation, function, and bone formation [8]. This is also true for TGF-ß signaling, as members of this super family also play a central role in bone metabolism [22]. TGIF, as a possible actuator, may be of particular importance in both signaling pathways [23, 24]. Loss of TGIF expression might consequently lead to their deregulation not only in osteoblasts, but also in cancer cells, promoting tumor progression and metastasis in BC. In this context, the role of TGIF in Wnt signaling might also connect estrogen and TGIF signaling in osteoblast [25] with possible effects on BC metastasis.

The GAIN study examined the use of adjuvant ibandronate [7] and is to our knowledge so far the only study on adjuvant bisphosphonate use with tumor tissue available. Here, we retrospectively investigated 1197 BC samples prospectively collected from this trial for TGIF immunostaining. Our results suggest that moderate to high TGIF expression is a common feature of breast cancer cells and that weak or absent TGIF immunoreactivity is linked to tumor progression.

A moderate/strong expression of TGIF correlated with favorable prognostic parameters like smaller tumor size, a well-differentiated phenotype and a HR-positive status. Moreover, we found a significant association between weak/absent TGIF expression and an adverse clinical outcome with shorter DFS and OS. Stratifying into molecular subgroups, we showed a significant favorable prognostic effect of TGIF expression on DFS in ER-positive and HER2-negative tumors and, consequently, in non-TNBC cases in univariate analysis with significant interaction only for HER2-negative BC subgroup. Regarding the treatment with ibandronate, the prognostic value of moderate/strong TGIF expression for both DFS and OS did not reach statistical significance in the individual groups with or without ibandronate treatment but showed a similar tendency towards improvement in both examined groups.

However, regarding bone metastases that occurred as first site of relapse, we were not able to demonstrate a prognostic influence of TGIF in the total cohort.

Currently, the most exciting potential of new biomarkers is the prediction of response to targeted therapy. In case of TGIF and its role as regulator in osteoblast function, it could be hypothesized that patients with elevated TGIF expression might respond to treatment with bisphosphonate, such as ibandronate. However, we could not observe a significant predictive effect of TGIF here either (data not shown).

Until now, there are only few data regarding the role of TGIF in breast cancer cells in clinical studies. Recently, Zhang et al reported that in TNBC, elevated levels of TGIF correlate with high Wnt signaling and poor survival of BC patients, which is opposite to our results [26]. However, this study was conducted in only 173 BC patients which were subjected to various treatment regimens [26]. Interestingly, the BC subgroups (ER-positive, HER2-negative) which exhibit a strong favorable prognostic impact of TGIF expression in our study are also reported to preferentially metastasize to the bone [27]. Yet, regarding time to bone metastasis, our results did not demonstrate an impact of TGIF expression on the development of skeletal metastases, but only on DFS and OS in non-TNBC patients.

One potential drawback of our study is the small number of cases with bone metastasis documented as first site of relapse. This could explain the lack of correlation between TGIF and bone metastases in general or with the use of adjuvant bisphosphonates. However, data from von Minckwitz et al. concluded that, 2 years of adjuvant treatment with ibandronate after dose-dense chemotherapy in the GAIN trial had acceptable adverse effects but did not improve survival in patients with high-risk breast cancer. Post hoc subgroup analyses support the hypothesis that adjuvant bisphosphonate activity is restricted to patients with low estrogen levels, either because of medical ovarian suppression or definite menopause. Future meta-analyses on an individual patient data level may reliably reveal subgroups in which this approach has the best efficacy [7]. Patient follow-up in the GAIN trial was based on clinical routine which does not recommend routine bone scans or other radiology assessments. This approach is in line with clinical guidelines and also common practice in most adjuvant BC trials. Therefore, the endpoint of bone disease as first site of metastatic spread might be difficult to assess in current clinical trials since patients could also develop other metastases before they show symptoms of their disease. However, a major strength of this work is the large cohort of this study with over 2994 patients, of whom 1380 samples were used for the TMA and 1197 cases were evaluable for TGIF expression. The uniform treatment of the patients with dose-dense chemotherapy which is still considered as standard in the adjuvant setting, and our findings support a biologic role of TGIF in BC patients treated in this context. To our knowledge, the clinical study GAIN is the only trial examining bisphosphonates or not that has FFPE samples available.

Previous studies suggest that TGIF might be associated with the initiation and progression of lung [14, 15], gastric [12] or colorectal cancer [13], which is opposite to our present results on breast cancer. Yet, those studies were largely performed by in vitro experiments, and only small numbers of human tumor tissue samples were analyzed, mostly without follow-up data. Our data generated on a high number of well-characterized breast cancer patients clearly show that, although TGIF is expressed in most tumor samples, low/absent expression of this protein is significantly associated with a less differentiated phenotype and poor outcome. This suggests differences in TGIF function in various tumor entities. Among breast cancer patients, differences were also found according to molecular subtypes: while negative/low TGIF expression correlates with shorter DFS and OS in ER-positive BC, it lacks any prognostic significance in TNBC, suggesting an interference of TGIF and ER signaling, which was already described in osteoblasts [25]. Furthermore, a significant interaction was observed only between high TGIF expression and HER2-negative BC. The nature of this interaction in BC cells should be further analyzed in experimental systems to elucidate the exact role and underlying mechanism of TGIF.

## Conclusions

In this study we have demonstrated that weak or absent TGIF expression in tumor cells is significantly associated with BC progression, especially in luminal carcinomas. However, comprehensive analysis of in vivo models is necessary, particularly regarding the mechanistic regulation of TGIF.

## Supplementary Information

**Additional file 1.**

**Additional file 2.**

## Data Availability

The data that support the findings of this study are available from the corresponding author upon reasonable request.

## Abbreviations

BC: Breast cancer
BCBM: Breast Cancer bone metastasis
DFS: Disease-free survival
HR: Hormone receptor
OS: Overall survival
TGIF: TGFB-induced factor homeobox 1
TNBC: Triple-negative breast cancer
TTBM: Time to bone metastasis
SRE: skeletal-related events

## References

[1] Bray F, Ferlay J, Soerjomataram I, Siegel RL, Torre LA, Jemal A (2018). Global cancer statistics 2018: GLOBOCAN estimates of incidence and mortality worldwide for 36 cancers in 185 countries. CA Cancer J Clin.

[2] Weigelt B, Peterse JL, van’t Veer LJ (2005). Breast cancer metastasis: markers and models. Nat Rev Cancer.

[3] Harbeck N, Gnant M (2017). Breast cancer. Lancet.

[4] O'Carrigan B, Wong MH, Willson ML, Stockler MR, Pavlakis N, Goodwin A (2017). Bisphosphonates and other bone agents for breast cancer. Cochrane Database Syst Rev.

[5] Menshawy A, Mattar O, Abdulkarim A, Kasem S, Nasreldin N, Menshawy E, et al. Denosumab versus bisphosphonates in patients with advanced cancers-related bone metastasis: systematic review and meta-analysis of randomized controlled trials. Support Care Cancer. 2018;26(4):1029–38. 10.1007/s00520-018-4060-1. 10.1007/s00520-018-4060-1 29387997

[6] Clemons M, Russell K, Costa L, Addison CL (2012). Adjuvant bisphosphonate treatment for breast cancer: why did something so elegant become so complicated?. Breast Cancer Res Treat.

[7] von Minckwitz G, Mobus V, Schneeweiss A, Huober J, Thomssen C, Untch M, et al. German adjuvant intergroup node-positive study: a phase III trial to compare oral ibandronate versus observation in patients with high-risk early breast cancer. J Clin Oncol. 2013;31(28):3531–9. 10.1200/JCO.2012.47.2167. 10.1200/JCO.2012.47.2167 23980081

[8] Baron R, Kneissel M (2013). WNT signaling in bone homeostasis and disease: from human mutations to treatments. Nat Med.

[9] Saito H, Gasser A, Bolamperti S, Maeda M, Matthies L, Jahn K, et al. TG-interacting factor 1 (Tgif1)-deficiency attenuates bone remodeling and blunts the anabolic response to parathyroid hormone. Nat Commun. 2019;10(1):1354. 10.1038/s41467-019-08778-x. 10.1038/s41467-019-08778-x PMC6430773 30902975

[10] Bertolino E, Reimund B, Wildt-Perinic D, Clerc RG (1995). A novel homeobox protein which recognizes a TGT core and functionally interferes with a retinoid-responsive motif. J Biol Chem.

[11] Burglin TR (1997). Analysis of TALE superclass homeobox genes (MEIS, PBC, KNOX, Iroquois, TGIF) reveals a novel domain conserved between plants and animals. Nucleic Acids Res.

[12] Hu ZL, Wen JF, Xiao DS, Zhen H, Fu CY (2005). Effects of transforming growth interacting factor on biological behaviors of gastric carcinoma cells. World J Gastroenterol.

[13] Wang JL, Qi Z, Li YH, Zhao HM, Chen YG, Fu W (2017). TGFbeta induced factor homeobox 1 promotes colorectal cancer development through activating Wnt/beta-catenin signaling. Oncotarget.

[14] Wang Y, Wang H, Gao H, Xu B, Zhai W, Li J, et al. Elevated expression of TGIF is involved in lung carcinogenesis. Tumour Biol. 2015;36(12):9223–31. 10.1007/s13277-015-3615-8. 10.1007/s13277-015-3615-8 26091794

[15] Xiang G, Yi Y, Weiwei H, Weiming W (2015). TGIF1 promoted the growth and migration of cancer cells in nonsmall cell lung cancer. Tumour Biol.

[16] McShane LM, Altman DG, Sauerbrei W, Taube SE, Gion M, Clark GM, et al. REporting recommendations for tumor MARKer prognostic studies (REMARK). Nat Clin Pract Urol. 2005;2(8):416–22. 16482653

[17] Remmele W, Stegner HE (1987). Recommendation for uniform definition of an immunoreactive score (IRS) for immunohistochemical estrogen receptor detection (ER-ICA) in breast cancer tissue. Pathologe.

[18] Gray RJ (1988). A class of K-sample tests for comparing the cumulative incidence of a competing risk. Ann Stat.

[19] Fine JP, Gray RJ (1999). A proportional hazards model for the subdistribution of a competing risk. J Am Stat Assoc.

[20] Mobus V, von Minckwitz G, Jackisch C, Luck HJ, Schneeweiss A, Tesch H, et al. German Adjuvant Intergroup Node-positive Study (GAIN): a phase III trial comparing two dose-dense regimens (iddEPC versus ddEC-PwX) in high-risk early breast cancer patients. Ann Oncol. 2017;28(8):1803–10. 10.1093/annonc/mdx203. 10.1093/annonc/mdx203 28459941

[21] Wilson C, Bell R, Hinsley S, Marshall H, Brown J, Cameron D, et al. Adjuvant zoledronic acid reduces fractures in breast cancer patients; an AZURE (BIG 01/04) study. Eur J Cancer. 2018;94:70–8. 10.1016/j.ejca.2018.02.004. 10.1016/j.ejca.2018.02.004 29544162

[22] Early Breast Cancer Trialists’ Collaborative G (2015). Adjuvant bisphosphonate treatment in early breast cancer: meta-analyses of individual patient data from randomised trials. Lancet.

[23] Wotton D, Lo RS, Swaby LA, Massague J (1999). Multiple modes of repression by the Smad transcriptional corepressor TGIF. J Biol Chem.

[24] Wotton D, Massague J (2001). Smad transcriptional corepressors in TGF beta family signaling. Curr Top Microbiol Immunol.

[25] Galea GL, Meakin LB, Sugiyama T, Zebda N, Sunters A, Taipaleenmaki H, et al. Estrogen receptor alpha mediates proliferation of osteoblastic cells stimulated by estrogen and mechanical strain, but their acute down-regulation of the Wnt antagonist Sost is mediated by estrogen receptor beta. J Biol Chem. 2013;288(13):9035–48. 10.1074/jbc.M112.405456. 10.1074/jbc.M112.405456 PMC3610976 23362266

[26] Zhang MZ, Ferrigno O, Wang Z, Ohnishi M, Prunier C, Levy L, et al. TGIF governs a feed-forward network that empowers Wnt signaling to drive mammary tumorigenesis. Cancer Cell. 2015;27(4):547–60. 10.1016/j.ccell.2015.03.002. 10.1016/j.ccell.2015.03.002 PMC4398914 25873176

[27] Wu Q, Li J, Zhu S, Wu J, Chen C, Liu Q, et al. Breast cancer subtypes predict the preferential site of distant metastases: a SEER based study. Oncotarget. 2017;8(17):27990–6. 10.18632/oncotarget.15856. 10.18632/oncotarget.15856 PMC5438624 28427196

