# Correction to: TGFB-induced factor homeobox 1 (TGIF) expression in breast cancer

**DOI:** 10.1186/s12885-021-08754-z

**Published:** 2021-09-15

**Authors:** Christine Stürken, Volker Möbus, Karin Milde-Langosch, Sabine Schmatloch, Peter A. Fasching, Josef Rüschoff, Elmar Stickeler, Rolf-Peter Henke, Carsten Denkert, Lars Hanker, Christian Schem, Valentina Vladimirova, Thomas Karn, Valentina Nekljudova, Claus-Henning Köhne, Frederik Marmé, Udo Schumacher, Sibylle Loibl, Volkmar Müller

**Affiliations:** 1 Institute of Anatomy and Experimental Morphology, University Medical Center Hamburg-Eppendorf, Martinistrasse 52, 20246 Hamburg, Germany; 2 Klinik für Gynäkologie und Geburtshilfe, Klinikum Frankfurt Höchst GmbH, Frankfurt am Main, Germany; 3 Department of Gynecology, University Medical Center Hamburg-Eppendorf, Martinistrasse 52, 20246 Hamburg, Germany; 4 Elisabeth Krankenhaus Kassel, Kassel, Germany; 5 Universitätsklinikum Erlangen, Erlangen, Germany; 6 Uniklinik RWTH Aachen, Aachen, Germany; 7 Klinikum Oldenburg, Oldenburg, Germany; 8 Institute of Pathology, Philipps-University Marburg and University Hospital Marburg (UKGM), Marburg, Germany; 9 Department of Gynecology and Obstetrics, University Hospital of Schleswig-Holstein, Campus Lübeck, Kiel, Germany; 10 Mammazentrum Hamburg, Hamburg, Germany; 11 German Breast Group, Neu-Isenburg, Germany; 12 Goethe University, Frankfurt, Germany; 13 Medizinische Fakultät Mannheim, Universität Heidelberg, Universitätsfrauenklinik Mannheim, Mannheim, Germany

**Correction to: BMC Cancer 21, 920 (2021)**

**https://doi.org/10.1186/s12885-021-08656-0**

Following publication of the original article [1], the authors noticed an incorrect affiliation for Christine Stürken and Udo Schumacher. The correct affiliations are as follows:

Christine Stürken

**Institute of Anatomy and Experimental Morphology, University Medical Center Hamburg-Eppendorf, Martinistrasse 52, 20246 Hamburg, Germany.**

Udo Schumacher

**Institute of Anatomy and Experimental Morphology, University Medical Center Hamburg-Eppendorf, Martinistrasse 52, 20246 Hamburg, Germany.**

The affiliations have been correctly published in this correction and the original article [1] has been updated.
